# Decolorization of Distillery Spent Wash Using Biopolymer Synthesized by *Pseudomonas aeruginosa* Isolated from Tannery Effluent

**DOI:** 10.1155/2015/195879

**Published:** 2015-10-01

**Authors:** Charles David, M. Arivazhagan, M. N. Balamurali, Dhivya Shanmugarajan

**Affiliations:** ^1^Environmental Biotechnology Research Laboratory, Department of Chemical Engineering, National Institute of Technology, Tiruchirappalli, Tamil Nadu 620 015, India; ^2^Department of Biotechnology, Madha Engineering College, Chennai, Tamil Nadu 600 069, India; ^3^Biotechnology Division, Asthagiri Herbal Research Foundation, Chennai, Tamil Nadu 600 096, India

## Abstract

A bacterial strain was isolated from tannery effluent which can tolerate high concentrations of potassium dichromate up to 1000 ppm. The isolated microorganism was identified as *Pseudomonas aeruginosa* by performing biochemical tests and molecular characterization. In the presence of excess of carbohydrate source, which is a physiological stress, this strain produces Polyhydroxybutyrate (PHB). This intracellular polymer, which is synthesized, is primarily a product of carbon assimilation and is employed by microorganisms as an energy storage molecule to be metabolized when other common energy sources are limitedly available. Efforts were taken to check whether the PHB has any positive effect on spent wash decolorization. When a combination of PHB and the isolated bacterial culture was added to spent wash, a maximum color removal of 92.77% was found which was comparatively higher than the color removed when the spent wash was treated individually with the PHB and *Pseudomonas aeruginosa*. PHB behaved as a support material for the bacteria to bind to it and thus develops biofilm, which is one of the natural physiological growth forms of microorganisms. The bacterial growth in the biofilm and the polymer together acted in synergy, adsorbing and coagulating the pollutants in the form of color pigments.

## 1. Introduction

Molasses based distillery effluent contains intense quantities of recalcitrant pollutants in the form of dark colored organic pollutants. The intense color is due to the presence of a dark brown, acidic melanoidin pigment [1]. Melanoidin are a group of polymeric compounds which are a product of the Maillard reaction, a nonenzymatic reaction between sugars and amino compounds [2, 3]. The empirical formula of melanoidin is C_17-18_H_26-27_O_10_N [4]. These antioxidant and recalcitrant polymers cannot be easily degraded by conventional biological treatment methods, namely, anaerobic digestion (biomethanation), anaerobic lagoons, and activated sludge process [5, 6]. When the untreated effluent gets released into surface water resources, the dark coloration of melanoidin hinders the penetration of sunlight into the water, thereby decreasing the photosynthetic activity and eventually affecting the life of aquatic microbiome [7]. Moreover, the high concentrations of chemical oxygen demand (COD), biochemical oxygen demand (BOD), and biodegradable organic materials, namely, carbohydrate, lignin, hemicellulose, dextrins, organic acids, and obnoxious odor [8, 9], were also present in the spent wash effluent. Hence, disposing untreated spent wash effluent into the environment is unsafe to the ecosystem due to high pollution potential [10]. Physicochemical treatment methods involve adsorption, coagulation and flocculation, electrocoagulation, advanced oxidation, ozonation, membrane filtration, and evaporation. Adsorption and charge neutralization is one of the major physical-chemical treatment methods employed for removing pollutants and color.

Biopolymer belongs to the polyesters class which is produced by microorganisms. The types of aliphatic polyesters are Polyhydroxyalkanoates (PHA), Polycaprolactone (PCL), and Polylactic acid (PLA). Polyhydroxyalkanoates (PHA) are hydroxyacid polyesters that are synthesized and accumulated as intracellular granules by a wide variety of bacteria [11]. Of the big family of PHAs, Polyhydroxybutyrate (PHB) is the most widespread and well characterized [11]. PHB has aroused much interest in industry and research as a biocompatible, biodegradable, thermoplastic, and piezoelectric polymer with potential applications in medical, agricultural, and marine fields. Generally, the production of PHB is enhanced when a suitable carbon source is available in excess, but the cellular growth is limited by another nutrient such as nitrogen or phosphorus [11, 12]. Some bacteria can accumulate up to 60–80% of their weight as PHB [13]. Of the big family of PHA, a homopolymer of 3-hydroxybutyrate, poly-3-hydroxybutyrate (PHB), is the most widespread and the best characterized. The polyester PHB is synthesized and accumulated as intracellular granules by a wide variety of bacteria. It is generally accepted that microorganisms isolated from a natural environment are poor in nutrient sources and these microorganisms exhibit higher survival abilities than those living in the alimentary tract of higher organisms. It is well recognized that this lipid inclusion is accumulated by bacteria as they enter the stationary phase of growth to be used later as an internal reserve of carbon and energy. Among the factors restricting the economy of PHB production is the cost of the carbon source. Hence, there arises a lookout for a suitable and inexpensive carbon source for bulk production of microbial PHB.

As PHB is produced from the microorganisms, they are well supported in the development of bacterial biofilm which is one of the natural physiological growth forms for microorganisms over these polymer structures. By using this biopolymer as support material, the biofilm can be enhanced to develop well and it is interesting to use a microbial film immobilized on a micro-carrier surface for the production of a wide variety of biochemicals that can be utilized for other different purposes. One of the natural physiological growth forms for a microorganism is a biofilm, in which the microbial community is attached to a solid surface. From the biotechnological point of view, it is interesting to use a microbial film immobilized on a surface as a support material for the production of a wide variety of biochemicals that can be utilized for different purposes [14].

The objective of this study focuses on isolation, identification, and characterization of chromium tolerant bacterial strain from tannery effluent. Lab scale production of PHB using the isolated bacterial strain uses spent wash as the sole carbon source. Degradation of organic pollutants in terms of spent wash color uses PHB produced using the isolated bacterial strain.

## 2. Materials and Methods

### 2.1. Collection of Tannery Effluent Sample

The tannery effluent sample was collected from Pallavaram Tanners Industrial Effluent Treatment Co. (PTIETC) located near Chromepet, Chennai, India. This facility treats 3000 m^3^/day of tannery effluent from the leather processing industrial cluster located nearby. Sample from the activated sludge tank were aseptically collected in sterilized glass bottles and transported to the laboratory and stored in the refrigerator at 4°C.

### 2.2. Collection of Distillery Effluent Sample

The distillery effluent sample was collected from Trichy Distilleries and Chemicals Limited (TDCL), located near the city of Tiruchirappalli, India. The collected effluent was immediately brought to the laboratory and stored in the refrigerator at 4°C [15, 16] until further use in order to avoid any deterioration in the physicochemical property of the spent wash.

### 2.3. Isolation of Metal Tolerant Bacterial Strain from Tannery Effluent

The metal tolerant bacterial strain was isolated by selection pressure method [17]. Chromium in the form of potassium dichromate (K_2_Cr_2_O_7_) was added in varying concentrations of 10–2000 ppm to sterile nutrient agar (pH 7.0). The plates were loaded with 500 *μ*L of raw effluent and the media was cast by pour-plate method. The colonies developed were counted after 3–7 days of incubation at 28°C. It is possible that some of the organisms die off due to pour-plate method. Consequently, the numbers of Cr (VI) resistant bacterial colonies able to grow were viewed on relative or comparative basis. The increasing concentration of chromium in the growth medium was given as a stress to resist the growth of the microorganisms. The strain capable of growing at maximum concentration was isolated. The isolated bacterial strain was identified with reference to* Bergey's Manual of Determinative Bacteriology* [18].

### 2.4. Molecular Characterization

The 24-hour fresh* Pseudomonas* sp. culture was taken for genomic DNA extraction based on isolation protocol described by Pitcher et al. [19]. The extracted DNA sample was run on 1% agarose gel with 1 k standard marker acquired from Bangalore Genei Private Limited, India. The universal primers were used to amplify the 16S rRNA gene region. The PCR amplification of 20 *μ*L reaction mixture containing 1 *μ*L of the template, primers: 2 *μ*L of forward primer, U3 (5′AGTGCCAGCAGCCGCGGTAA3′), 2 *μ*L of reverse primer, U4 (5′AGGCCCGGGAACGTATTCAC3′) [20], 12 *μ*L of assay buffer, 1 *μ*L of* Taq* DNA polymerase, and 2 *μ*L of dNTP mix. The amplification was carried out in Thermal Cycler for 35 cycles using the following reaction conditions,* namely,* initial denaturation of DNA at 95°C for 5 minutes, denaturation of DNA at 95°C for 30 seconds, primer annealing at 45°C for 90 seconds, and primer extension at 72°C for 1 minute. The amplified PCR product was mixed with 2 *μ*L of gel loading buffer and 1% agarose gel was cast. The samples were loaded along with 2 *μ*L of 1 kb DNA ladder as a molecular marker. The gel was run and examined on a UV transilluminator to visualize the bands. PCR products were purified by using EZ-10 spin column PCR purification kit and it was sequenced.

### 2.5. Production of PHB

**Figure 1:**
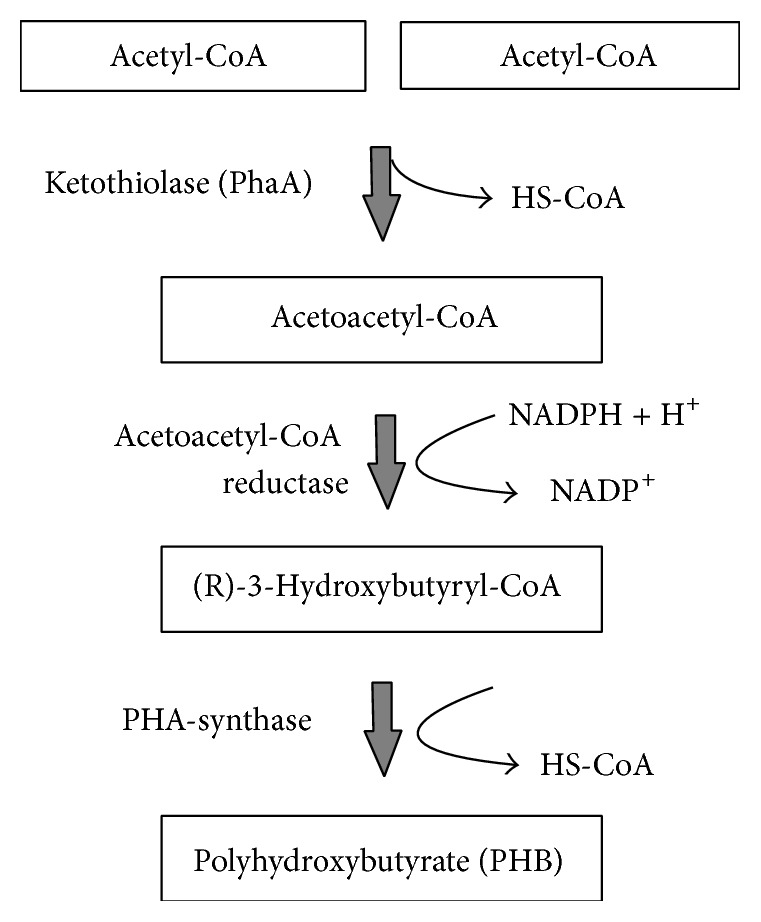
Biosynthetic pathway of Polyhydroxybutyrate (PHB).

Vincent [21] proposed the composition of minerals and nutrients to be used in yeast extract mannitol (YEM) broth for the production of PHB. The isolated and identified bacterial strain from tannery effluent was used for the production of PHB. Yeast extract mannitol (YEM) broth (g/L) consists of following ingredients: mannitol, 10 g; KH_2_PO_4_, 0.5 g; MgSO_4_·7H_2_O, 0.2 g; NaCl, 0.1 g; tryptone, 2.5 g; peptone, 2.5 g; yeast extract, 2.5 g. The pH of the medium was adjusted to 7.0 with dilute HCl. The batch production of PHB was carried out in 250 mL Erlenmeyer flasks containing 100 mL of culture medium. The temperature was maintained at 30°C and the culture was agitated at 110 rpm. The production medium was inoculated with a loopful of isolated bacterial culture. The biosynthetic pathway of PHB is shown in Figure 1.

### 2.6. Harvesting and Assay of PHB

**Figure 2:**
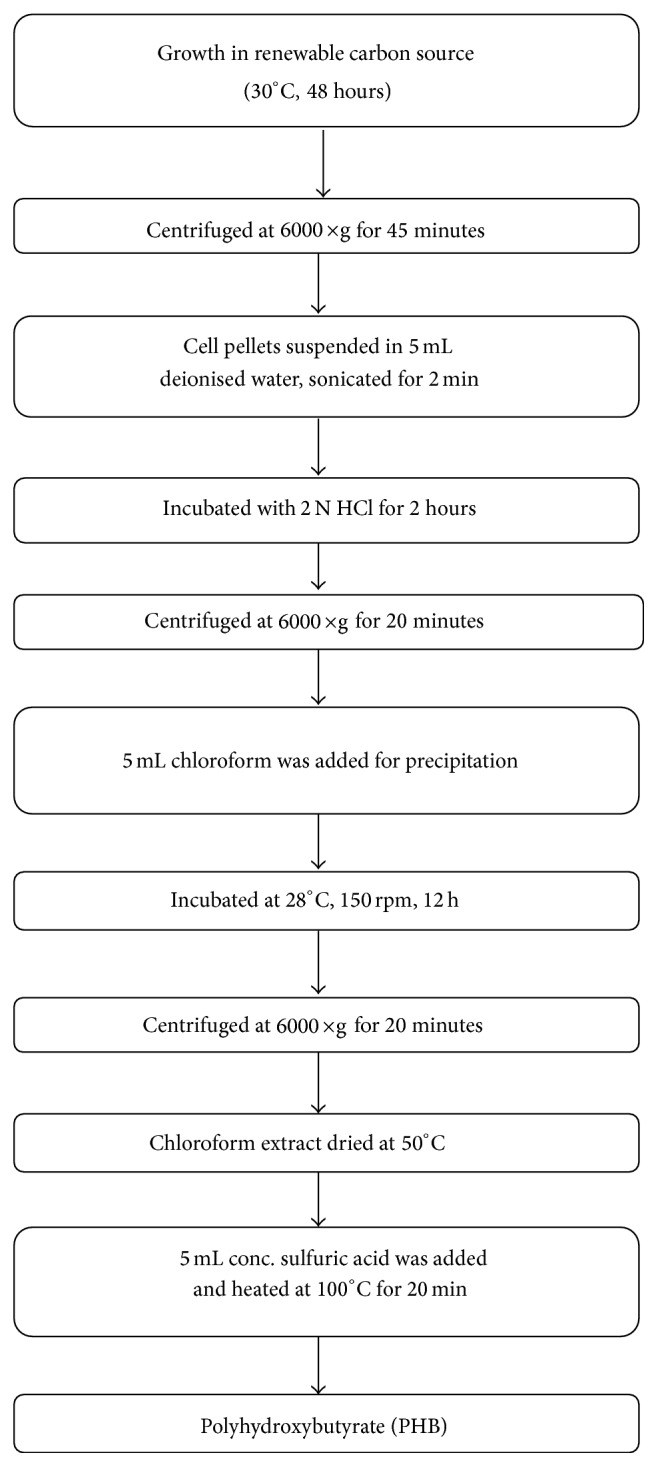
Schematic flow diagram representing harvesting and purification of PHB.

The isolated, metal tolerant bacterial strain was cultured in YEM broth at 30°C for 48 hours in an incubator shaker. Cultures at stationary phase of growth were centrifuged at 6000 ×g for 45 min. The cell-free supernatant was discarded. The cell pellets were suspended in 5 mL of deionised water and homogenized for 2 min in a sonicator bath. To 2 mL of the cell suspension, 2 mL of 2 N HCl was added and boiled for 120 min in a water bath. The tubes were centrifuged at 6000 ×g for 20 min. To obtain precipitate, 5 mL of chloroform was added. The test tubes containing the suspension were left overnight at 28°C on a shaker at 150 rpm. The contents of the test tubes were centrifuged at 6000 ×g for 20 minutes and 0.1 mL of chloroform extract was dried at 50°C. About 5 mL of concentrated sulfuric acid was added and heated at 100°C in water bath for 20 min. After cooling to room temperature, the amount of PHB was determined using UV-Vis spectrophotometer at a corresponding wavelength of 235 nm. The schematic step-wise procedure for PHB harvesting is shown in Figure 2.

### 2.7. Determination of Dry Cell Weight

The total dry weight (total biomass) was determined by harvesting, washing, drying to constant volume, and weighing. The non-PHB dry weight (non-PHB biomass) was calculated from the total dry weight and the PHB content using the following equation:

(1) Non-PHB dry weight=total dry weight×100−%PHB100.

### 2.8. Effect of Different Carbon Sources on PHB Production

The usage of mannitol in YEM medium broth was replaced by other carbon sources such as glucose, fructose, dextrose, and sucrose in the growth medium. Peptone and tryptone were kept as constant nitrogen sources. Based on the well-known fact that molasses is rich in carbon source and inexpensive, trials were performed in which the expensive carbon source has been replaced by inexpensive molasses. The PHB yield for different carbon sources and molasses was determined.

### 2.9. Spent Wash Decolorization Studies

The batch color removal experiments were performed in Erlenmeyer flasks (250 mL volume) containing 100 mL of raw spent wash. An appropriate dosage of as-synthesized PHB and 48-hour-old bacterial culture was added as listed below:

1. 2 mL of* Pseudomonas aeruginosa* culture.
2. 2 mL of PHB synthesized using the isolated strain.
3. 2 mL (1 : 1 ratio) of PHB and* Pseudomonas aeruginosa* culture.

The batch vessels were shifted to an incubator shaker and the flasks were mildly shaken at 50 rpm. Color reduction was monitored for 120 h. Aliquots of samples were withdrawn and centrifuged at 10000 ×g for 10 min to remove the suspended particles. Color removal was measured at a characteristic wavelength of 475 nm using UV-Visible spectrophotometer (Spectroquant, Pharo 300, Merck).

The color removal efficiency was calculated by

(2) $$\begin{array}{c} \text{Color removed}\left(\;\%\;\right)=\frac{C_{0}-C_{t}}{C_{0}}\times 100, \end{array}$$

where *C*_0_ and *C*_*t*_ are the initial absorbance and absorbance at time *t* for spent wash effluent at a characteristic wavelength of 475 nm [22, 23].

## 3. Results and Discussion

### 3.1. Isolation of Metal Tolerant Strain

**Figure 3:**
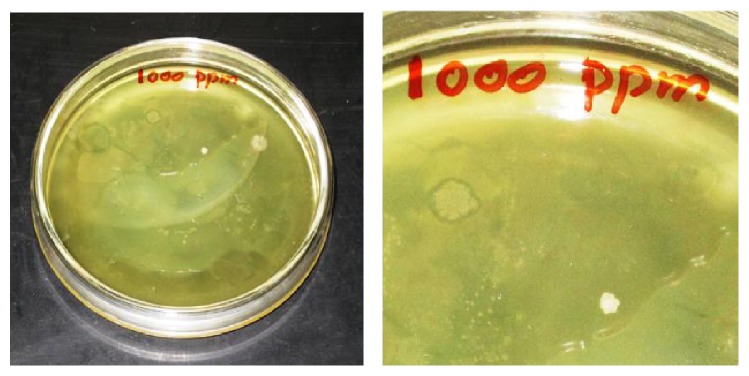
Microbial colony developed at 1000 ppm of K_2_Cr_2_O_7_ dosage concentration.

By selection pressure method, the most tolerant bacterial strain was isolated from tannery effluent. This strain was found to tolerate a maximum concentration of 1000 ppm (1000 *μ*g/mL) of K_2_Cr_2_O_7_, when cultured in nutrient agar media containing K_2_Cr_2_O_7_ as shown in Figure 3. This method is to enhance the selection pressure, thereby reducing the number of surviving species, and only to obtain the organism that can withstand such high concentration (1000 ppm) of K_2_Cr_2_O_7_. At lower concentrations, numerous well developed colonies were visualized. But, at 1000 ppm of concentration, only very few numbers of colonies were formed. These highly tolerant colonies were subcultured and preserved for identification, molecular characterization of the strain, and production of secondary metabolite.

### 3.2. Identification of Isolated Bacterial Strain

**Figure 4:**
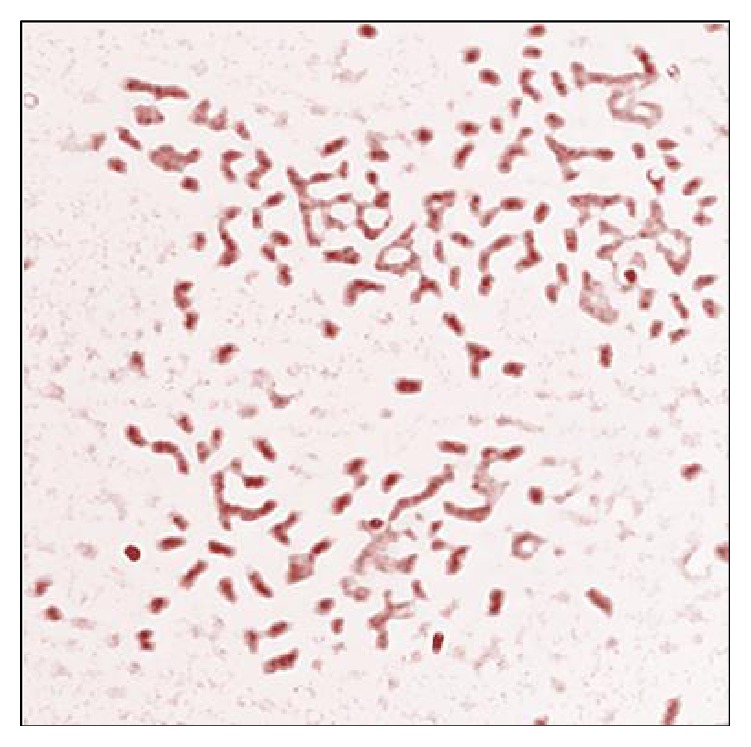
Gram's staining showing Gram-negative rods of isolated* Pseudomonas aeruginosa*.

The microorganism isolated by the selection pressure method was identified by performing morphological, microbial, and biochemical tests and the results were compared with* Bergey's Manual of Determinative Bacteriology*. The colonies formed by the isolated strain were irregular circular in shape, with flat colony elevation, with uneven or rough colony margin, and dull white to mild beige in color. The microorganisms were identified to be Gram-negative motile rods as shown in Figure 4. The strain isolated tested positive in catalase test, due to the rapid evolution of gas bubbles, when a drop of H_2_O_2_ was placed on the bacterial colony, showing that there was an evolution of oxygen and the strain is aerobic. When subjected to oxidase test, the result was positive. This is due to the formation of dark blue, purple color which indicates the presence of cytochrome c oxidase. Indole test gave a negative result as there was no formation of the cherry red colored ring when Kovac's reagent was added to the incubated culture. Phenol red test result was negative as the isolated strain cannot ferment any of the sugars like glucose, sucrose, or lactose. So there was neither a change in color nor formation and collection of gas inside the inverted Durham's tubes. The result was methyl red negative upon performing methyl red test as there was no red color formation upon addition of methyl red indicator which denotes the fact that the pH remains above 6.0. Formation of colorless colonies was seen when they were grown on EMB and MacConkey agar plates. This is due to the reason that the organism cannot ferment lactose sugars. The biochemical tests and the corresponding results are tabulated in Table 1.

### 3.3. Molecular Characterization

**Figure 5:**
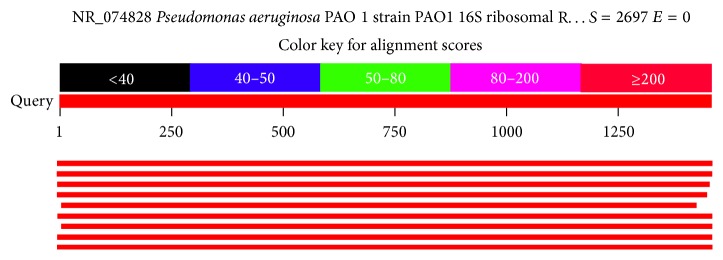
BLAST results for the isolated bacterial strain* Pseudomonas aeruginosa*.

The PCR sequenced product was identified using Bioinformatics tool, BLAST, and* Pseudomonas aeruginosa* gene for 16S rRNA, partial sequence with 98% query coverage and 99% identity with expected value of zero was found. This confirmed that the organism is* Pseudomonas aeruginosa*. The sequence is given as follows and the BLAST results are shown in Figure 5:

GCAGGCCTAACACATGCAAGTCGAGCGGATGAAGGGAGCTTGCTCCTGGATTCAGCGGCGGACGGGTGAGTAATGCCTAGGAATCTGCCTGGTAGTGGGGGATAACGTCCGGAAACGGGCGCTAATACCGCATACGTCCTGAGGGAGAAAGTGGGGGATCTTCGGACCTCACGCTATCAGATGAGCCTAGGTCGGATTAGCTAGTTGGTGGGGTAAAGGCCTACCAAGGCGACGATCCGTAACTGGTCTGAGAGGATGATCAGTCACACTGGAACTGAGACACGGTCCAGACTCCTACGGGAGGCAGCAGTGGGGAATATTGGACAATGGGCGAAAGCCTGATCCAGCCATGCCGCGTGTGTGAAGAAGGTCTTCGGATTGTAAAGCACTTTAAGTTGGGAGGAAGGGCAGTAAGTTAATACCTTGCTGTTTTGACGTTACCAACAGAATAAGCACCGGCTAACTTCGTGCCAGCAGCCGCGGTAATACGAAGGGTGCAAGCGTTAATCGGAATTACTGGGCGTAAAGCGCGCGTAGGTGGTTCAGCAAGTTGGATGTGAAATCCCCGGGCTCAACCTGGGAACTGCATCCAAAACTACTGAGCTAGAGTACGGTAGAGGGTGGTGGAATTTCCTGTGTAGCGGTGAAATGCGTAGATATAGGAAGGAACACCAGTGGCGAAGGCGACCACCTGGACTGATACTGACACTGAGGTGCGAAAGCGTGGGGAGCAAACAGGATTAGATACCCTGGTAGTCCACGCCGTAAACGATGTCGACTAGCCGTTGGGATCCTTGAGATCTTAGTGGCGCAGCTAACGCGATAAGTCGACCGCCTGGGGAGTACGGCCGCAAGGTTAAAACTCAAATGAATTGACGGGGGCCCGCACAAGCGGTGGAGCATGTGGTTTAATTCGAAGCAACGCGAAGAACCTTACCTGGCCTTGACATGCTGAGAACTTTCCAGAGATGGATTGGTGCCTTCGGGAACTCAGACACAGGTGCTGCATGGCTGTCGTCAGCTCGTGTCGTGAGATGTTGGGTTAAGTCCCGTAACGAGCGCAACCCTTGTCCTTAGTTACCAGCACCTCGGGTGGGCACTCTAAGGAGACTGCCGGTGACAAACCGGAGGAAGGTGGGGATGACGTCAAGTCATCATGGCCCTTACGGCCAGGGCTACACACGTGCTACAATGGTCGGTACAAAGGGTTGCCAAGCCGCGAGGTGGAGCTAATCCCATAAAACCGATCGTAGTCCGGATCGCAGTCTGCAACTCGACTGCGTGAAGTCGGAATCGCTAGTAATCGTGAATCAGAATGTCACGGTGAATACGTTCCCGGGCCTTGTACACACCGCCCGTCACACCATGGGAGTGGGTTGCTCCAGAAGTAGCTAGTCTAACCGCAAGGGGGACGGTTACCACGGAGTGATTCATGACTGGGGTGAAGTCGTAACAAGGTA

### 3.4. Production of Polyhydroxybutyrate (PHB) by Using the Isolated Bacterial Strain

*Pseudomonas aeruginosa*, Gram-negative motile rod shaped bacteria, were able to synthesize Polyhydroxybutyrate (PHB) as an intracellular secondary metabolite which is a resultant product due to the physiological stress occurring due to the availability of excess amount of carbon source and limited availability of other minerals especially phosphate or nitrogen. It has been suggested that ammonia limited cultures of* Pseudomonas aeruginosa* were unable to regulate fully the rate at which they take up glucose, particularly when growing at the low availability of minerals. As a result, they form copious amounts of exopolysaccharide, both to overcome the potentially deleterious osmotic effects of accumulating surplus intracellular metabolites and to consume some of the surplus ATP generated by the oxidation of these metabolites [24–26]. However, unlike exopolysaccharide, PHB is an intracellular product and therefore additionally provides a means of storing excess carbon and reducing power for future use [27]. In this context, it is interesting to note that* Pseudomonas aeruginosa* can synthesize PHB or other Polyhydroxyalkanoates, exopolysaccharide, and/or various organic acids as alternative products, after losing its ability to make exopolysaccharide or PHB, respectively, by following natural strain degeneration or mutagenesis [25, 28].

### 3.5. Effect of Different Carbon Sources on PHB Production

The yield of PHB based on various carbon sources was studied and the values along with standard deviation is tabulated in Table 2. When the carbon source, mannitol, in the YEM medium was replaced by molasses, a maximum yield of 70% PHB was obtained. This research finding was a success as the molasses were used and the cost due to the use of mannitol can also be avoided. This is an economical initiative of using molasses as a source of carbon in the growth medium of* Pseudomonas aeruginosa*.

The Dunnett's multiple comparison test was used to find the statistical significance of the various carbon sources in comparison with the control (YEM). In Table 3, the values with *p* < 0.05 are considered significant with symbol *∗* indicating mild significance and symbol *∗∗∗∗* indicating more significance in comparison with the control medium.

### 3.6. Effect of Time on PHB Production

**Figure 6:**
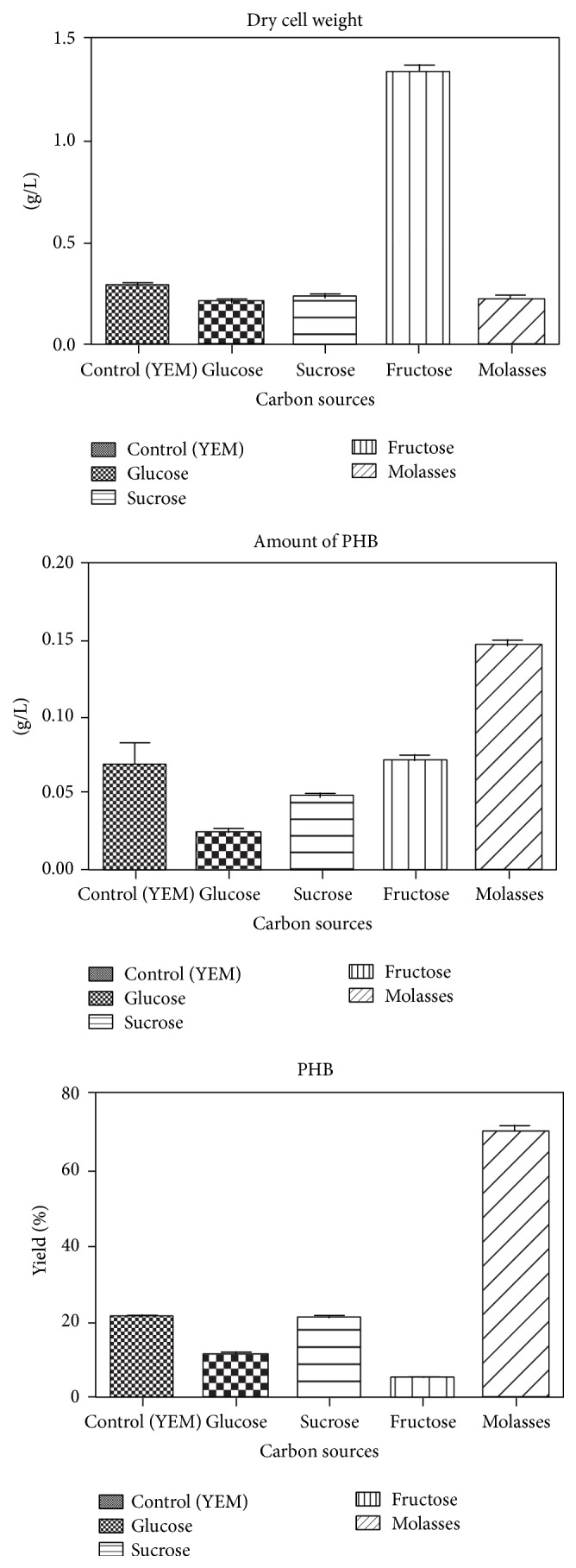
Bar charts with standard deviation values for dry cell weight, amount of PHB, and % yield of PHB.

It was found that when molasses were used as the carbon source in YEM medium instead of mannitol, at the end of 48 hours, the PHB yield was 70%. After 48 hours of incubation, there was a decrease in the PHB yield and increase in the viscosity of the medium. The increase in the viscosity of the growth medium resulted in a limited oxygen transfer rate and caused the fall of PHB synthesis and accumulation inside the bacterial cells. The PHB yield decreased to 32% after 72 hours of incubation and 18% after 120 hours of incubation. Even the dry cell weight was increased up to 120 hours. The decrease in the PHB content explained that the bacteria have used the produced PHB as a source of carbon to survive due to the unavailability of the carbon source. The %PHB yield along with the standard deviation values has been plotted as shown in Figure 6.

### 3.7. Spent Wash Decolorization Study

**Figure 7:**
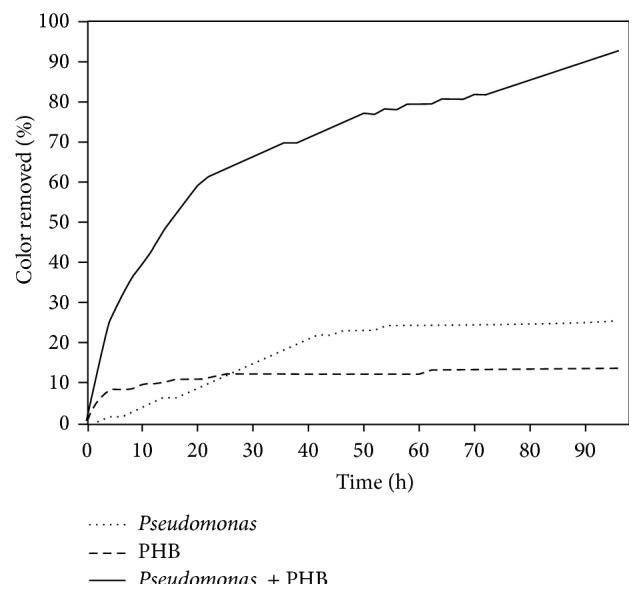
Effect of time on % color removal by microbial culture, PHB, and combination of microbial culture and PHB.

This initiative of testing the effect of the isolated bacterial culture and the as-synthesized PHB on spent wash decolorization was performed as a trial and very positive and welcoming results were obtained as shown in Figure 7. At the end of five-day batch study, a combination consisting of 2 mL (1 : 1 ratio) of PHB and* Pseudomonas aeruginosa* culture was able to achieve 92.77% spent wash color removal, whereas there was only a minimal color reduction (25.30% and 13.58%) resulting when the spent wash was treated with microorganism and PHB individually. The increased color removal was due to the phenomenon that the Gram-negative bacteria,* Pseudomonas aeruginosa,* possess negative surface charge. When these bacterial cultures were added to the effluent along with the PHB, the bacteria bind to the PHB, hence forming a biofilm, and thereby also act as an ion exchange that attracts the suspended organic particles to get bound to the biofilm. This biofilm acts as a support material and favors a suitable condition for the further growth and development of the bacteria. Thus, the synergic actions of the PHB and the microorganism were found to be the most capable of performing spent wash decolorization.

### 3.8. Research Outcome

The positive results of this research could lead to a more advanced technique and application where the microbially produced PHB can be used as a nanobiomaterial possessing tunable properties with a focused application for binding and removal of heavy metals from aqueous industrial effluents. As the synthesis of biopolymer relies on a principle of single phase transition, scaling up the production process could be of a less intensive task, hence providing an ecofriendly technology for pollutant removal.

## 4. Conclusion

In this research paper, bacterial strain possessing tolerance to high concentrations of chromium was isolated from tannery effluent. The isolated strain was identified as* Pseudomonas aeruginosa* by biochemical and molecular characterization. Efforts were taken to synthesize Polyhydroxybutyrate (PHB) using the isolated bacterial strain. Mannitol, an expensive carbon source for the bacterial growth culture media, was replaced with inexpensive molasses. The exopolysaccharides accumulated by the bacterial cells were harvested and separated. Optimization of suitable quantities of as-synthesized PHB and microbial culture was tested to evaluate the color removal efficiency. The results showed that* Pseudomonas aeruginosa* exhibited a synergistic effect in combination (1 : 1 ratio) with the biopolymer towards spent wash decolorization. Lab scale optimization experiments resulted in 92.77% removal of spent wash color after 96 hours of treatment, whereas there was only a limited color reduction (25.30% and 13.58%) observed when the same concentration and volume of spent wash was treated with* Pseudomonas aeruginosa* culture and PHB individually.

## Figures and Tables

**Table 1:** Biochemical and morphological characterization of isolated bacterial strain.

Biochemical test	Result	Morphology	Result
Gram staining	−	Colony shape	Irregular round
Catalase test	+	Colony elevation	Flat
Oxidase test	+	Colony size (mm)	2.5
Indole test	−	Colony margin	Serrated
Phenol red test	−	Colony color	Dull white
Methyl red test	−	Motility	Motile
Growth on EMB agar	−	Cell shape	Rod
Growth on MacConkey agar	−		

**Table 2:** Effect of various carbon sources on PHB production with standard deviation values.

Carbon source	Dry cell weight (g/L)	SD	Amount of PHB (g/L)	SD	PHB yield (%)	SD
Glucose	0.22	0.007	0.026	0.0007	11.82	0.049
Sucrose	0.23	0.01	0.049	0.0008	21.30	0.141
Fructose	1.32	0.0282	0.072	0.0021	5.45	0.219
Molasses	0.21	0.0131	0.147	0.0014	70.0	0.707
Control (YEM)	0.28	0.0141	0.060	0.0135	21.43	0.0636

SD: standard deviation.

**Table 3:** Effect of various carbon sources on PHB production with standard deviation values.

Dunnett's multiple comparison test	Significant	Summary	Adjusted *p* value
Dry cell weight			
Control (YEM) versus glucose	Yes	*∗*	0.0178
Control (YEM) versus sucrose	No	ns	0.0683
Control (YEM) versus fructose	Yes	*∗∗∗∗*	<0.0001
Control (YEM) versus molasses	Yes	*∗*	0.0235

Amount of PHB			
Control (YEM) versus glucose	Yes	*∗∗*	0.0029
Control (YEM) versus sucrose	No	ns	0.0682
Control (YEM) versus fructose	No	ns	0.9459
Control (YEM) versus molasses	Yes	*∗∗∗*	0.0002

% yield of PHB			
Control (YEM) versus glucose	Yes	*∗∗∗∗*	<0.0001
Control (YEM) versus sucrose	No	ns	>0.9999
Control (YEM) versus fructose	Yes	*∗∗∗∗*	<0.0001
Control (YEM) versus molasses	Yes	*∗∗∗∗*	<0.0001

ns: not significant.
